# Upon the Composition of the Semen in Old Men

**Published:** 1853-07

**Authors:** A. Duplay


### ANATOMY AND PHYSIOLOGY.

*Upon the composition of the semen in old men.* By Dr. A. Duplay.—Bérard, Burdach, Muller, Longet, state, that spermatozoa are absent in the seminal secretion of the aged; Wagner alone asserts, that, in men of seventy to eighty years, these bodies are found in the vesiculæ seminales, if not in the testicle. Facts tend to prove that the fecundating power is retained up to the age of 100. The author investigated the semen in fifty-one subjects, after death caused by various diseases. In its physical properties the semen was sometimes of yellow color, as in the adult; generally of a paler straw hue, or almost colorless, milky or creamy. In twelve cases it was thick; but, in the greater number, it was serous, though mixed with the usual mucous secretion from the vesiculæ seminales. In most cases the vesiculæ seminales were full of fluid; in one only were they atrophied. The semen in the vasa deferentia was always darker-colored than that in the vesiculæ.

In thirty-seven cases the author remarked the presence of spermatozoa; in fourteen he failed in detecting them. In twenty-seven cases they were such as are usually seen in the healthy adult; in the rest the tail was shorter, and the head sometimes separated. Once he saw a number of crystals, whose nature he could not investigate. Considerable variety existed in their proportion.

In twenty-six cases, spermatozoa were found along the whole tract of the spermatic passages; three times only in the vas deferens; once only in the fluid of the vesiculæ seminales; seven times in the vesicula seminalis of the left side only. In the latter cases there existed mostly degenerations of the testicle, or obliteration of some part of the seminal duct. Secretion of semen continued, however, in cases where there was considerable atrophy of the testicle; the highest weight of which was 11·98 grammes; the lowest 4·50 grammes. The tissue of the testicle was always normal; the epididymis contained occasionally cysts. In four cases there was hydrocele. In the fourteen cases where there were no spermatozoa, the subjects had passed the age of seventy; but there were no special changes discoverable in the glands. The secretion of semen, concludes Dr. Duplay, continues in old men, but in a less degree; their semen contains spermatozoa even when the person is beyond the age for fecundation. The cause, therefore of want of power to effect this end cannot be explained upon the idea of the deficiency of spermatozoa.—*Medical Times, from Arch. Gen. Dec.* 1852.

